# Reasons for incomplete STI vaccination among men who have sex with men in an English sexual health service

**DOI:** 10.1177/09564624231165078

**Published:** 2023-03-21

**Authors:** Heather L Armstrong, Clare Scholfield, Ynez Symonds, Tom Nadarzynski, Cynthia A Graham

**Affiliations:** 1 Solent NHS Sexual Health Service, Southampton, UK; 2 7423 University of Southampton, Southampton, UK; 3 247209 University of Westminster, London, UK

**Keywords:** Men who have sex with men, Vaccination, HPV, Hepatitis

## Abstract

**Background:**

In England, vaccination for human papillomavirus, hepatitis A, and hepatitis B is recommended for men who have sex with men (MSM). However, uptake is sub-optimal and some men do not complete all recommended vaccine doses. This service evaluation aimed to explore reasons for lack of uptake for each of these vaccines among MSM in one English sexual health service and to inform improvements in service delivery to increase full dose completion rates.

**Methods:**

MSM, ≥18 years, who had previously attended NHS Solent Sexual Health for at least one vaccination, and who had not completed the full dosing regimen for at least one of these vaccines, were invited to participate in an anonymous, online survey between 14/12/2020-11/04/2021.

**Results:**

Among 246 MSM (*M* = 42.1 years), the most common reason for non-vaccination was that participants thought it was unneeded and had not been recommended by a doctor or healthcare provider. None reported vaccine hesitancy. Likewise, the most common reasons for vaccination were doctor/healthcare provider recommendation (51.7–65.6%) and self-protection (60.9–68.1%). The most common reason for not having completed the full course of vaccination was being unaware that the next dose was due (30.0–37.8%). Many participants who had not completed vaccination indicated that a doctor/healthcare provider recommendation would be a motivating factor and that reminder messages and being able to book subsequent appointments in advance would facilitate vaccination.

**Conclusions:**

Sexual health clinicians should be encouraged to discuss STI vaccination with MSM and services should explore possibilities to improve ease and access to vaccine appointments to increase uptake and completion rates.

## Introduction

Human papillomavirus (HPV), Hepatitis A (HAV), and Hepatitis (HBV) are three viruses which can be transmitted through sexual contact, but which can also be effectively prevented through vaccination.^1–4^ In England, vaccination is available free of charge through the National Health Service (NHS) for those who meet eligibility criteria, including men who have sex with men (MSM).^3,4^ However, to be fully effective, a full course of vaccine doses is required. As such, it is important to better understand why some eligible MSM either are not vaccinated, or do not complete the full recommended vaccine course.

Among MSM, HPV infection can cause anogenital warts and some strains, if left untreated, can cause oropharyngeal, genital, and anal cancers.^5–7^ Genital cancers can include penile cancer among cisgender MSM and vulvovaginal and cervical cancer among transgender MSM.^4^ HAV and HBV are liver infections which can be transmitted sexually or through injection drug use. While most cases clear on their own, symptoms can persist and lead to liver damage, cancer, or failure.^4^

Effective vaccinations for the prevention of HPV, HAV, and HBV are available. In the UK, school-based HPV vaccination programs have been in place for girls since September 2008 but for boys only since 2019.^8^ As such, vaccination is needed for older individuals who were not offered vaccination while in school. HPV vaccination requires two doses of vaccine, at least 6 months apart^4^ and among MSM, vaccination has been shown to be cost-effective and to reduce HPV infection and associated cancers.^9,10^ HAV vaccination is not routinely offered in the UK^3^ and while HBV vaccination is now standard for all babies born since 2017,^4^ older individuals will likely not have been vaccinated. A combined HAV/HBV vaccine is available for those at higher risk, including MSM, people with multiple sexual partners, and people who inject drugs. Individuals should receive three doses, typically at 0, 1, and 6-months intervals.^4^ The vaccines may also be given independently, with a primary dose and booster 6–12 months later recommended for HAV and three doses required for HBV.^3,4^ When fully administered, HAV and HBV vaccination is up to 95% effective.^1–3^

Previous research among MSM in England has found that most MSM have poor knowledge about HPV and associated cancers and that most are also unaware of the availability or need for vaccination.^11,12^ In a qualitative study of 33 MSM, while MSM did not perceive themselves to be at risk for HPV, nearly all would accept the vaccine if offered by a healthcare professional.^11^ A larger online survey of 1508 MSM found that less than 20% knew about HPV, but that 55% would be willing to ask for the HPV vaccine and 89% would accept it if offered by a healthcare professional.^12^ Additional barriers to vaccination identified by MSM across these studies included accessing sexual health services and discussing same-sex behaviour with healthcare professionals; some were also unsure about vaccine efficacy.^11,12^ Further research from Scotland demonstrated that, in the year following the implementation of a national HPV immunisation program of MSM in July 2017, 64% of eligible MSM attending sexual health clinics received at least one vaccine dose.^13^ More recently, a systematic review and meta-analysis of 78 studies examined acceptability, uptake, and completion rates among MSM for HPV, HAV, HBV, and meningitis C vaccination.^14^ The authors concluded that while levels of acceptability were high, uptake and completion rates were lower than recommended in epidemiological models. As such, increased uptake and completion rates would be of benefit both to individual MSM and the larger population.

This service evaluation aimed to explore reasons for the lack of uptake of each of the three STI vaccines among MSM in one English sexual health service and to inform improvements in service delivery to increase full dose completion rates. Primarily we wanted to answer the question: why do some MSM receive their first dose of vaccination for HPV, HAV, or HBV, but then not continue to completion of the dosing schedule?

## Methods

All study participants were recruited through Solent NHS Sexual Health Services in Southampton, Hampshire, England. Individuals who had previously attended Solent Sexual Health for at least one dose of HPV, HAV, or HBV vaccination, and who had not completed the full dosing regimen for at least one of these vaccines, were identified through the patient information system database (INFORM). Additional eligibility criteria included male sex, being 18 years of age or older, having had male sexual partners, and having a valid mobile number, as recorded in INFORM.

Data were collected between 14 December 2020–11 April 2021. Eligible participants were sent an initial text message in December 2020 inviting them to complete the survey. The message included a link to access the participant information sheet and survey. Between February and March 2021, eligible participants were sent a second reminder text with the link and were asked to complete the survey if they had not previously done so. All participants completed the anonymous survey online.

Upon clicking on the study link, individuals were taken to the participant information sheet. Consent was assumed if participants clicked the “Next” button at the bottom of the participant information sheet to access the survey. Participants were informed that they could stop the survey at any time by closing the browser window. The survey was anonymous and confidential, was designed to take 5–10 min to complete, and responses were not linked to any patient information. Participants did not receive any compensation for participation. All study procedures were approved by Solent Sexual Health Clinical Governance and the University of Southampton Ethics Review board (ERGO number 57903).

The survey consisted of four sections: demographics, followed by one section each for HPV vaccination, HAV vaccination, and HBV vaccination. Demographics included age, gender, sex assigned at birth, area of residence, ethnicity, sexual orientation, and gender of sexual partners (Table 1). At the beginning of each vaccination section, participants were asked whether they had received that vaccine (yes/no/I don’t know) and subsequent questions presented depended on their response. Participants who indicated that they had not received the vaccine were asked to indicate why and given a short list of primary reasons. If ‘yes’ was indicated for some of these reasons, further related options were then presented to allow for greater specification (see Table 2 for a full list of possible reasons). Participants were also able to indicate “I had another reason” and provide a response via text. Participants who indicated they had received a particular vaccine were asked how many doses they had received, when they had received their last dose, why they received the vaccine, and any challenges they faced in attending the clinic for vaccination. Additionally, participants who indicated that they had not received all the required doses of the vaccine, or who were unsure of how many doses they had received, were asked why they had not yet had their next dose (with primary and sub-reasons as described above) and their intention to receive future doses. All participants were asked if there was anything that would motivate them to receive the vaccine (or the next dose of the vaccine, as relevant), and if there was anything that would make it easier for them to attend the clinic for vaccination in the future. The questions were repeated for each of the three vaccines. Following completion of the survey, participants were shown a debriefing form with information on how to book a vaccine and contact information for sexual health services.

**Table 1.** Sample demographics (*n* = 246).

Demographic	*n*	%
Gender	—	—
Male	241	98.0
Non-binary	4	1.6
Transgender	1	0.4
Location	—	—
Southampton	69	28.0
Portsmouth	59	24.0
Isle of Wight	5	2.0
Elsewhere in Hampshire	81	32.9
England, outside of Hampshire	25	10.2
Somewhere else	7	2.8
Ethnicity	—	—
White	230	93.9
Black	6	2.4
Mixed	4	1.6
Any other ethnic group (e.g., Asian, Arab, Hispanic/Latino)	5	2.0
Sexual orientation	—	—
Gay	184	74.8
Straight/Heterosexual	31	12.6
Bisexual	24	9.8
Other (e.g., queer, pansexual)	7	2.8
Sexual partners	—	—
Male only	190	77.2
Female only	28	11.4
Male and female	19	7.7
Other genders	6	2.4
No sexual activity	3	1.2

**Table 2.** Reasons for not having had the HPV/HAV/HBV vaccines.

	First dose			Subsequent dose		
	HPV (*n* = 46)	HAV (*n* = 22)	HBV (*n* = 10)	HPV (*n* = 75)	HAV (*n* = 45)	HBV (*n* = 70)
	*N* (%)	*N* (%)	*N* (%)	*N* (%)	*N* (%)	*N* (%)
I do not think it is time for me to receive another dose	-	-	-	23 (30.7)	17 (37.8)	21 (30.0)
I do not think that I need it/another dose	18 (39.1)	11 (50.0)	6 (60.0)	5 (6.7)	10 (22.2)	20 (28.6)
I am at low risk for HPV/HepA/HepB	5 (10.9)	2 (9.1)	1 (10.0)	0 (0)	0 (0)	0 (0)
I have not had sex with anyone	1 (2.2)	0 (0)	0 (0)	0 (0)	0 (0)	0 (0)
I had not had sex with anyone who has HPV/HepA/HepB	2 (4.3)	2 (9.1)	2 (20.0)	0 (0)	2 (4.4)	0 (0)
My doctor or health care provider has not recommended the HPV/HepA/HepB vaccine	10 (21.7)	8 (36.4)	3 (30.0)	3 (4.0)	7 (15.6)	8 (11.4)
I have always had safer sex	1 (2.2)	0 (0)	0 (0)	0 (0)	0 (0)	0 (0)
I was healthy so did not need to get vaccinated	0 (0)	0 (0)	0 (0)	1 (1.3)	0 (0)	0 (0)
I was told that I didn’t need another dose	-	-	-	-	-	12 (17.1)
I found out I was already immune	-	-	-	-	-	2 (2.9)
I have been meaning to get it but I just haven’t done it yet	5 (10.9)	1 (4.5)	0 (0)	19 (25.3)	6 (13.3)	10 (14.3)
I decided I did not want to get the vaccine	2 (4.3)	2 (9.1)	2 (20.0)	1 (1.3)	1 (2.2)	0 (0)
I had difficulty getting the vaccine	9 (19.6)	2 (9.1)	1 (10.0)	17 (22.7)	3 (6.7)	7 (10.0)
I was unable to get an appointment for the vaccine	2 (4.3)	1 (4.5)	1 (10.0)	16 (21.3)	2 (4.4)	6 (8.6)
I attended clinic but the vaccine wasn’t available	2 (4.3)	0 (0)	0 (0)	2 (2.7)	1 (2.1)	0 (0)
I did not know where to get the vaccine	1 (2.2)	0 (0)	0 (0)	-	-	-
I could not take time off work to get to the clinic	0 (0)	0 (0)	0 (0)	0 (0)	0 (0)	0 (0)
I could not find childcare in order to go to the clinic	0 (0)	0 (0)	0 (0)	0 (0)	0 (0)	0 (0)
I did not have a way to get to the clinic	0 (0)	0 (0)	0 (0)	0 (0)	0 (0)	0 (0)
I did not know if the vaccine was covered by the NHS	4 (8.7)	1 (4.5)	0 (0)	-	-	-
I was worried about having the vaccine	0 (0)	0 (0)	0 (0)	0 (0)	0 (0)	0 (0)
I was worried about vaccine side effects	0 (0)	0 (0)	0 (0)	0 (0)	0 (0)	0 (0)
I was worried the vaccine could be dangerous	0 (0)	0 (0)	0 (0)	0 (0)	0 (0)	0 (0)
I don’t think the vaccine is effective	0 (0)	0 (0)	0 (0)	0 (0)	0 (0)	0 (0)
I did not want anyone to know that I was getting the vaccine	0 (0)	0 (0)	0 (0)	0 (0)	0 (0)	0 (0)
I was worried about being discriminated against	0 (0)	0 (0)	0 (0)	0 (0)	0 (0)	0 (0)
I was worried that someone might find out that I was going to the clinic	0 (0)	0 (0)	0 (0)	0 (0)	0 (0)	0 (0)
I had another reason	12 (26.1)	7 (31.8)	2 (20.0)	11 (14.7)	5 (11.1)	8 (11.4)
Unaware (e.g., of eligibility, of vaccine)	6 (13.1)	2 (9.1)	-	-	1 (2.1)	-
Age-related (e.g., “I thought I was too old”)	4 (8.7)	-	-	-	-	-
Mental health concerns/Needle phobia	1 (2.2)	1 (4.5)	-	-	-	2 (2.9)
Immune/have previously had	-	2 (9.1)	1 (10.0)	-	1 (2.1)	-
Not offered	-	1 (4.5)	-	-	-	-
Told to have in future	-	1 (4.5)	-	-	-	-
Forgot about second dose and wasn’t reminded	-	-	1 (10.0)	-	-	-
COVID-19 (e.g., unable to book, lockdown)	-	-	-	2 (2.7)	-	2 (2.9)
Reaction to previous dose	-	-	-	2 (2.7)	1 (2.1)	-
Unsure when next dose is due	-	-	-	2 (2.7)	-	-
Expected to/hadn’t been contacted to receive next dose	-	-	-	2 (2.7)	-	1 (1.4)
Vaccine supply issues	-	-	-	1 (1.3)	-	1 (1.4)
Moved out of area	-	-	-	1 (1.3)	-	-
Told another dose not needed	-	-	-	1 (1.3)	-	-
Unsure if they have had all doses	-	-	-	-	1 (2.1)	1 (1.4)

Data were analysed with SPSS v27 using descriptive statistics to explore reasons for having had and not had the vaccines, challenges faced, and motivations and facilitators for future vaccination.

## Results

3176 individuals meeting inclusion criteria as recorded in INFORM were sent a text message with the online study link. During the study period, there were 2348 visits to the survey website, of which 298 people attempted the questionnaire. After data cleaning for inclusion criteria and completion of the variables of interest, the final sample size was *n* = 246.

Participants had a mean age of 42.1 years (range 18–75, SD = 14.67). Most self-identified as male (98.0%) and reported currently living in Hampshire (87.0%). The majority also reported White ethnicity (93.9%), identified as gay (74.8%), and reported only male sex partners (77.2%). Full demographics are reported in Table 1.

Among all participants, 244 responded to questions about HPV vaccination, 224 to questions about HAV vaccination, and 220 to questions about HBV vaccination. The majority had received at least one dose of each vaccine (HPV: 128/244, 52.5%; HAV: 116/224, 51.8%; HBV: 138/220, 62.7%) but substantial proportions had not (HPV: 46/244, 18.9%; HAV: 22/224, 9.8%; HBV: 10/220, 4.5%), or were uncertain about their vaccine status (HPV: 70/244, 28.7%; HAV: 86/224, 38.4%; HBV: 72/220, 32.7%).

Of those who reported not having had a vaccine, when asked why, the most common reason reported was that they did not think it was needed (HPV: 18/46, 39.1%; HAV: 11/22, 50.0%; HBV: 6/10, 60.0%). Among these, many reported that vaccination had not been recommended by their doctor or healthcare provider (HPV: 10/18, 55.6%; HAV: 8/22, 36.4%; HBV: 3/10, 30.0%). Further, among those who had not received the HPV vaccine, 19.6% (9/46) reported difficulty attaining the vaccine, including not knowing that the vaccine was available without cost (*n* = 4), not being able to get an appointment (*n* = 2), and attending a clinic where the vaccine was not available (*n* = 2). Fewer participants reported difficulty attaining the HAV (*n* = 2, 9.1%) or HBV (*n* = 1, 10.0%) vaccines. Zero participants, across all vaccine types, reported not receiving the vaccine because they were worried (Table 2).

Of those who reported having received the HPV vaccine (*n* = 128), 22.8% reported one dose, 21.3% reported two doses, 41.7% reported three doses, and 14.2% were unsure how many doses they had received. Among those who reported having received the HAV vaccine (*n* = 116), 15.8% reported one dose, 60.5% reported two doses, and 23.7% were unsure. Lastly, of those who reported having received the HBV vaccine (*n* = 138), 11.8% reported one dose, 16.9% reported two doses, 48.5% reported three or more doses, and 22.8% were unsure how many doses they had received. Across all vaccines, the most reported reasons for vaccinations were the recommendation by a doctor or healthcare provider (HPV: 65.6%; HAV: 51.7%; HBV: 52.2%) and because they wanted to protect themselves (HPV: 60.9%; HAV: 64.7%; HBV: 68.1%) (Table 3).

**Table 3.** Reasons for vaccination among those who have been vaccinated.

	HPV (*n* = 128)		HepA (*n* = 116)		HepB(*n* = 138)	
	*N*	%	*N*	%	*N*	%
I want to protect myself from “genital warts and/or cancers caused by HPV”/HepA/HepB	78	60.9	75	64.7	94	68.1
I want to protect my sexual partners from “genital warts and/or cancers caused by HPV”/HepA/HepB	47	36.7	39	33.6	48	34.8
I believe I am at risk for “HPV and/or cancer caused by HPV”/HepA/HepB	26	20.3	21	18.1	32	23.2
I have had “HPV/genital warts”/HepA/HepB in the past	9	7.0	1	0.9	2	1.4
I believe “HPV and/or cancer caused by HPV”/HepA/HepB is a serious health concern	35	27.3	31	26.7	31	22.5
My doctor or healthcare provider recommended that I receive the vaccination	84	65.6	60	51.7	72	52.2
My sexual partner recommended that I receive the vaccination	3	2.3	5	4.3	4	2.9
A family member or friend recommended that I receive the vaccination	2	1.6	2	1.7	0	0.0
I have received previous doses of the vaccine	11	8.6	9	7.8	14	10.1
Other	-	-	7	6.0	7	5.1
Travel	-	-	3	2.6	1	0.7
Work-related reasons	-	-	2	1.7	4	2.9
Childhood vaccination program	-	-	1	0.9	1	0.7
“I had a low level”	-	-	-	-	1	0.7

Participants who had not completed the full course of vaccination (i.e., had received one or two doses for HPV or HBV, or one dose for HAV), or who were unsure how many doses they had received, were asked why they had not yet had their next dose and whether they intended to receive another dose (HPV: *n* = 75, HAV: *n* = 45, HBV: *n* = 70). The most common reason for not having had their next dose was that they did not think that it was time (HPV: 23/75, 30.7%; HAV: 17/45, 37.8%; HBV: 21/70, 30.0%). Additional common reasons included: for HPV, that they had been meaning to but just hadn’t done it yet (19/75, 25.3%), and for HAV and HBV: that they did not think that they needed another dose (HAV: 10/45, 22.2%; HBV: 20/70, 28.6%). Some participants also reported difficulty in getting the next dose (HPV: 17/75, 22.7%; HAV: 3/45, 6.7%; HBV: 7/70, 10.0%), most commonly that they were unable to get an appointment (Table 2). Most reported that they intended to get another dose of the vaccine (HPV: 58/73, 79.5%; HAV: 27/39, 69.2%; HBV: 39/59, 66.1%) and that they intended to do so within the next year (HPV: 69.9%; HAV: 59.0%; HBV: 54.2%).

Participants who reported having had a vaccine were asked if they faced any challenges attending the clinic to receive the vaccine (HPV: *n* = 128; HAV: *n* = 116; HBV: *n* = 138). The most common challenge was being unable to get an appointment (HPV: 18.8%; HAV: 7.8%; HBV: 10.1%) (Table 4).

**Table 4.** Reported challenges attending clinic.

	HPV (*n* = 128)		HepA (*n* = 116)		HepB (*n* = 138)	
	*N*	%	*N*	%	*N*	%
I was worried about having the vaccine	1	0.8	0	0.0	4	2.9
I did not know where to get the vaccine	3	2.3	3	2.6	2	1.4
I had trouble with transportation to get to the clinic	2	1.6	0	0.0	1	0.7
I had trouble getting time off work to get to the clinic	11	8.6	5	4.3	7	5.1
I had trouble finding childcare to get to the clinic	0	0.0	0	0.0	1	0.7
I was unable to get an appointment for the vaccine	17	13.3	9	7.8	14	10.1
I was worried that someone might find out that I was going to the clinic	6	4.7	3	2.6	3	2.2
I did not know if the vaccine was covered by the NHS	10	7.8	4	3.4	6	4.3
Other	17	19.5	10	11.6	10	7.2
COVID-19 (e.g., unable to book, lockdown, cancelled)	4	3.1	-	-	1	0.7
Trouble getting appointment	2	1.6	-	-	-	-
Staff/Clinic challenges (e.g., staff unsure how many doses participant had)	2	1.6	-	-	-	-
Unaware more than one dose needed	1	0.8	-	-	-	-
Unsure when to get second dose	1	0.8	-	-	-	-
Cannot book in advance	1	0.8	-	-	1	0.7
Received vaccine at another location	-	-	3	2.6	-	-
Side effects	-	-	1	0.9	-	-
Can’t get it done locally	-	-	1	0.9	-	-
Did not know immunity level	-	-	-	-	1	0.7
Problems developing immunity	-	-	-	-	1	0.7
Missed does	-	-	-	-	1	0.7
Works away	-	-	-	-	1	0.7

Finally, participants who had not received a vaccine, not completed a full course of vaccination, or who were unsure of how many doses they had received, were asked what would motivate them to get the vaccine (or their next dose) and if there was anything that would make it easier for them to attend clinics for vaccination in the future (HPV: *n* = 192; HAV: *n* = 174; HBV: *n* = 180). A large majority indicated that they would get the vaccine if a doctor or healthcare provider recommended it (HPV: 80.2%; HAV: 67.2%; HBV: 61.7%). Many participants also indicated that they would like to receive a SMS/text reminder (45.6–58.3%), email (28.5–56.3%), or phone call (11.5–16.1%), and that they would like to be able to schedule their appointment in advance (27.8–37.0%) (Table 5).

**Table 5.** Motivations and facilitators to attend clinic for future vaccine.

	HPV (*n* = 192)		HepA (*n* = 174)		HepB (*n* = 180)	
	*n*	%	*n*	%	*n*	%
Motivations	—	—	—	—	—	—
I would get the vaccine if my doctor or healthcare provider recommended it	154	80.2	117	67.2	111	61.7
I would get the vaccine if my sexual partner recommended it	27	14.1	17	9.8	22	12.2
I would get the vaccine if a family member recommended it	19	9.9	11	6.3	15	8.3
I would get the vaccine if a friend recommended it	19	9.9	14	8.0	14	7.8
I would get the vaccine if I become sexually active	5	2.6	11	6.3	5	2.8
I would get the vaccine if I start having sex with more partners	17	8.9	13	7.5	13	7.2
I would get the vaccine if I had more information about it	20	10.4	18	10.3	12	6.7
I would get the vaccine if I knew what the side effects would be	2	1.0	3	1.7	2	1.1
Not applicable. I do not need the HPV/HepA/HepB vaccine	7	3.6	7	4.0	5	2.8
Not applicable. I have completed all doses of the HPV/HepA/HepB vaccine	2	1.0	10	5.7	11	6.1
Facilitators	—	—	—	—	—	—
I would like to receive a reminder email	84	56.3	67	28.5	63	35.0
I would like to receive a reminder phone call	31	16.1	20	11.5	24	13.3
I would like to receive a reminder SMS/text message	112	58.3	88	50.6	82	45.6
I would like to be able to schedule my next appointment in advance	71	37.0	55	31.6	50	27.8
I would prefer an appointment first thing in the morning	12	6.3	10	5.7	10	5.6
I would prefer an appointment in the evening	24	12.5	21	12.5	20	11.1
I would prefer an appointment on the weekend	16	8.3	14	8.0	15	8.3
Not applicable. I do not need the HPV/HepA/HepB vaccine	13	6.8	9	5.2	7	3.9
Not applicable. I have completed all doses of the HPV/HepA/HepB vaccine	2	1.0	8	4.6	12	6.7
Other:	11	5.7	2	1.1	2	1.1
More availability to book online	3	1.6	-	-	-	-
More convenient location	2	1.0	-	-	-	-
More times to book evening/weekends	1	0.5	-	-	-	-
Fewer questions asked when booking	1	0.5	-	-	-	-
Able to receive vaccine at GP surgery	1	0.5	-	-	1	0.6
Integrate reminders with the MyGP app	1	0.5	-	-	-	-
Clearer information	1	0.5	-	-	-	-
Minimal wait time in clinic	1	0.5	-	-	-	-
Service should be proactive in reminding people when vaccines are due and offering appointments	-	-	1	0.6	-	-
Received conflicting information and lack of follow-up from service	-	-	-	-	1	0.6

## Discussion

The aim of this service evaluation was to better understand why some MSM receive their first vaccine dose for HPV, HAV, or HBV, but then do not complete the subsequent doses required to maximise vaccine efficiency. The most common reason reported for this across all vaccines was that individuals did not think that it was time for their next dose. Other common reasons included meaning to do so but had not done yet and thinking that another dose was not needed. A minority of participants reported difficulty in getting the next dose, primarily because they had been unable to get an appointment.

Inconsistent implementation of vaccination for HPV, HAV, and HBV can have widespread health and economic consequences for individuals, the general population, and public health services. An outbreak of HAV among MSM in England (July 2016-January 2018) resulted in 670 confirmed cases and healthcare costs were estimated around £1,500,000.^15^ Overall, our results indicate that sexual health services may be well positioned to help increase vaccine uptake and completion rates among MSM. Firstly, many of our participants reported being unaware that vaccination was recommended and that multiple doses were needed for full efficacy. Doctors and healthcare providers should discuss the benefits of vaccination with their patients and provide the needed education to increase awareness and understanding about dosing schedules and the need for multiple vaccine doses. Further, approximately one-third of men reported not being aware of their vaccine status. As such, doctors and healthcare providers should review vaccine status with their patients and routinely recommend vaccination and uptake of all doses for indicated patients.

A common reason for not having received any vaccination was that it had not been recommended by a doctor or healthcare provider and likewise, one of the most common reasons for vaccination was this recommendation. This was consistent across all types of vaccination. Our results demonstrate that MSM are largely willing to be vaccinated for these three STIs and would be more likely to complete the full dosing schedule with additional awareness, recommendations, and reminders from sexual health services. Indeed, when asked about potential facilitators for vaccination, the most commonly reported was a doctor or healthcare provider recommendation.

Some difficulty in attaining vaccines was also noted. Being unable to get an appointment was the most common reported challenge for all vaccination types. To a lesser extent, participants also reported difficulty in getting time off work to attend the appointment and several reported they were unaware that vaccination was covered by the NHS.

To increase vaccination and uptake of subsequent doses, participants reported that receiving a reminder by text or email, and to a lesser extent by phone, would be helpful. Participants also noted that they would like to be able to schedule their appointment in advance (not just on the day of), and some recommended being able to book subsequent doses during the initial vaccination. Similarly, participants recommended that appointments also be available to book in the evenings and weekends to accommodate different working schedules. Finally, participants noted that at the first vaccine appointment, it should be clearly communicated how many additional doses they will need. This was especially true for HAV/HBV vaccination as many participants who had not received the full course of doses reported being unaware that more were needed.

This evaluation had several limitations. First, participants were only invited to participate if they had previously attended Solent Sexual Health Service for STI vaccination but had not completed a full course of at least one of the STI vaccines. As such, participants in our sample were already in at least some contact with healthcare services and all had received at least one dose of one vaccine. Reasons for non-vaccination among men who have received no vaccines or who have had no contact with sexual health services may differ (e.g., higher vaccine hesitancy). Second, our response rate was low and those who completed our survey may differ from those who did not. For example, people who did not respond to our survey due to time constraints may find it more difficult to take time off work to attend the clinic, a response option which was selected by only a few of our participants. Third, our questionnaire was designed to be completed quickly and so we did not ask about some variables which may also affect vaccination knowledge such as level of education or frequency of attendance at sexual health services. Lastly, our data were collected before the monkeypox outbreak in Spring/Summer 2022. As such, we did not ask about vaccination for monkeypox. Vaccination for monkeypox is a single dose and so requires only one trip to a sexual health service. Potentially, discussion of monkeypox vaccination may encourage greater awareness of vaccination for other viruses which can be transmitted sexually. It may also be possible to provide the first dose of other STI vaccines at the same time as the monkeypox vaccine. Alternatively, a singular focus on monkeypox vaccination, due to heightened media coverage and disease severity, may adversely affect vaccination for other STIs which may be seen as less urgent or severe. Future research may wish to explore this in more detail.

In conclusion, sexual health services are well-positioned to increase awareness of vaccination needs among MSM and to promote and improve vaccine uptake. Vaccination status review and education about the benefits of vaccination and the need for multiple doses should be part of standard care when consulting with MSM and our results indicate that vaccine recommendations from healthcare providers would be highly effective. Sexual health services should also consider the feasibility of implementing additional strategies to improve uptake such as appointment scheduling procedures and SMS/email reminders.
